# The BEARD Score: A Practical Tool to Assess Beard Involvement in Alopecia Areata

**DOI:** 10.1111/ijd.70282

**Published:** 2026-01-14

**Authors:** Giuseppe Gallo, Nadia Sciamarrelli, Simone Ribero, Luca Mastorino, Pietro Quaglino, François Rosset

**Affiliations:** ^1^ Dermatology, Department of Medical Sciences University of Turin Turin Italy; ^2^ Department of Medical and Surgical Sciences Alma Mater Studiorum, University of Bologna Bologna Italy

**Keywords:** alopecia, alopecia areata, beard alopecia, BEARD score, hair

Beard involvement in alopecia areata (AA) is a common and often distressing manifestation in male patients, yet it remains largely under‐represented in the clinical outcome measures routinely used in practice and research. Existing scoring systems are either restricted to the scalp, such as the Severity of Alopecia Tool (SALT) [1], or require complex weighted calculations within composite indices like the Alopecia Areata Scalp Index (AASI) [2], leaving a gap for a rapid, visually‐based tool specifically designed for beard alopecia. Moreover, while patient‐reported outcomes, such as the Alopecia Areata Patient Priority Outcomes (AAPPO), Skindex‐16, and Hospital Anxiety and Depression Scale (HADS) [3], capture aspects of quality of life, they do not directly quantify beard involvement. Based on our clinical experience and the clear need for a dedicated assessment method, we propose the BEARD score (Beard Evaluation Alopecia Rating Degree), a simple, beard‐focused scoring system designed to quantify AA involvement in the beard area (Figure 1). The beard is anatomically divided into six regions, each equally weighted, adjusted for the average hair density typically present (Table 1).

**FIGURE 1:**
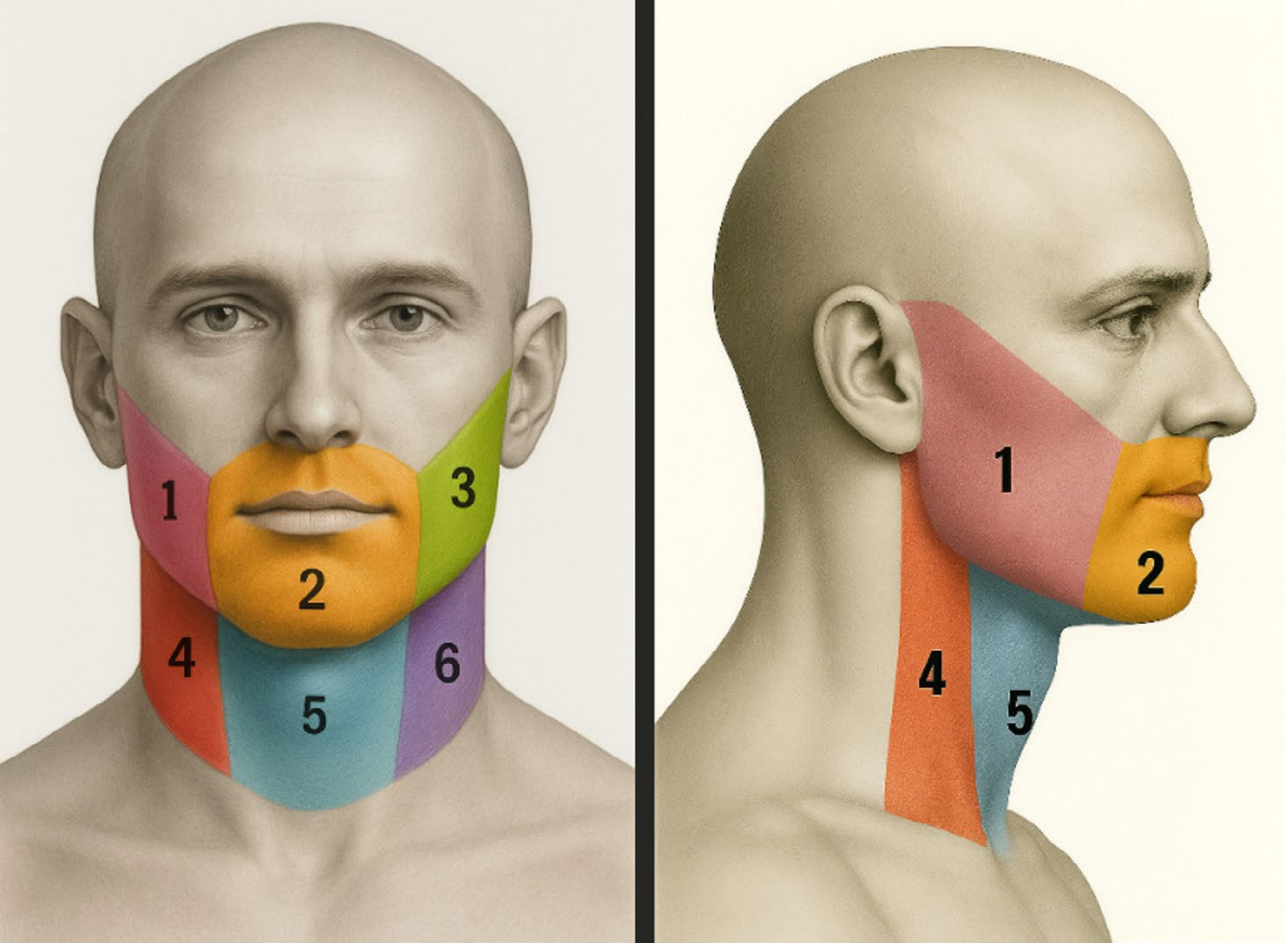
Visual representation of the six regions of the BEARD score. Frontal and lateral view.

**TABLE 1:** The BEARD score: proposed tool for assessing beard involvement in alopecia areata.

Area no.	Anatomic region	Score if involved
1	Right mandibular branch	1
2	Mustache area (above and below lips)	1
3	Left mandibular branch	1
4	Left lateral cervical region	1
5	Medial cervical region (incl. submental)	1
6	Right lateral cervical region	1

*Note:* Total score range: 0–6. Severity categories: mild: 1–2 areas involved (score 1–2); moderate: 3–4 areas involved (score 3–4); severe: 5–6 areas involved (score 5–6).

For each area with clinical involvement (defined practically as the presence of any hair loss compared to baseline or the contralateral unaffected area, regardless of severity), 1 point is assigned, yielding a total score ranging from 0 (no involvement) to 6 (full involvement).

To facilitate interpretation and clinical decision‐making, we propose the following categories:

– Mild: 1–2 areas involved;
– Moderate: 3–4 areas involved;
– Severe: 5–6 areas involved.

This approach offers several potential advantages: it is specific to beard involvement, easy to apply without complex calculations, and aligns more directly with the psychological impact frequently reported by patients with beard AA [4]. Furthermore, it could serve as a practical endpoint in clinical practice and in clinical trials targeting beard regrowth. Based on the literature reviewed, the patches initially appear along the lower jawline and on the neck, but no significant differences have been reported in the frequency of involvement among the six beard regions in alopecia areata [5]. However, data specifically addressing regional distribution within the beard remain limited [4, 5]. We recognize that the BEARD score is, at present, a theoretical proposal requiring validation. Future studies are needed to assess its interobserver reliability, sensitivity to change, and correlation with patient‐reported distress.

## Funding

The authors have nothing to report.

## Conflicts of Interest

The authors declare no conflicts of interest.

## Data Availability

The data that support the findings of this study are available from the corresponding author upon reasonable request.
